# Editorial: Comprehensive profiling cancer immunity with multimodal approaches for clinical management

**DOI:** 10.3389/fimmu.2024.1421576

**Published:** 2024-04-30

**Authors:** Geng Chen, Yi Shi, Wenming Xiao, David P. Kreil

**Affiliations:** ^1^ School of Life Sciences, East China Normal University, Shanghai, China; ^2^ Bio-X Institutes, Shanghai Jiao Tong University, Shanghai, China; ^3^ The Center for Drug Evaluation and Research, United States Food and Drug Administration, Silver Spring, MD, United States; ^4^ Department of Biotechnology, Boku University Vienna, Vienna, Austria

**Keywords:** cancer immunity, multimodal strategy, cancer management, multi-omics data analysis, cancer diagnosis, cancer prognosis, cancer treatment

Cancer is a multifactorial and highly heterogeneous disease, which predominantly arises from the confluence of genetic determinants, environmental exposures, and lifestyle factors (1–3). This complex etiology necessitates the employment of sophisticated diagnostic modalities and therapeutic interventions to effectively manage the disease. The immune system plays a central role in orchestrating both the oncogenic processes and the progression of cancer. An integrative approach that consolidates data from diverse domains, including but not limited to omics data sets, clinical parameters, and pathological profiles, offers unparalleled depth and breadth in deciphering and elucidating the complexities of cancer immunity, as compared to monodimensional methodologies (4–6).

In this Research Topic on Comprehensive profiling cancer immunity with multimodal approaches for clinical management, we aimed to collect novel researches that systematically investigates cancer immunity through the utilization of multimodal approaches for enhancing clinical management (**Figure 1**). A total of 8 papers were published in this collection, we provide a concise summary and discussion of the key findings from these studies in this editorial.

**Figure 1:**
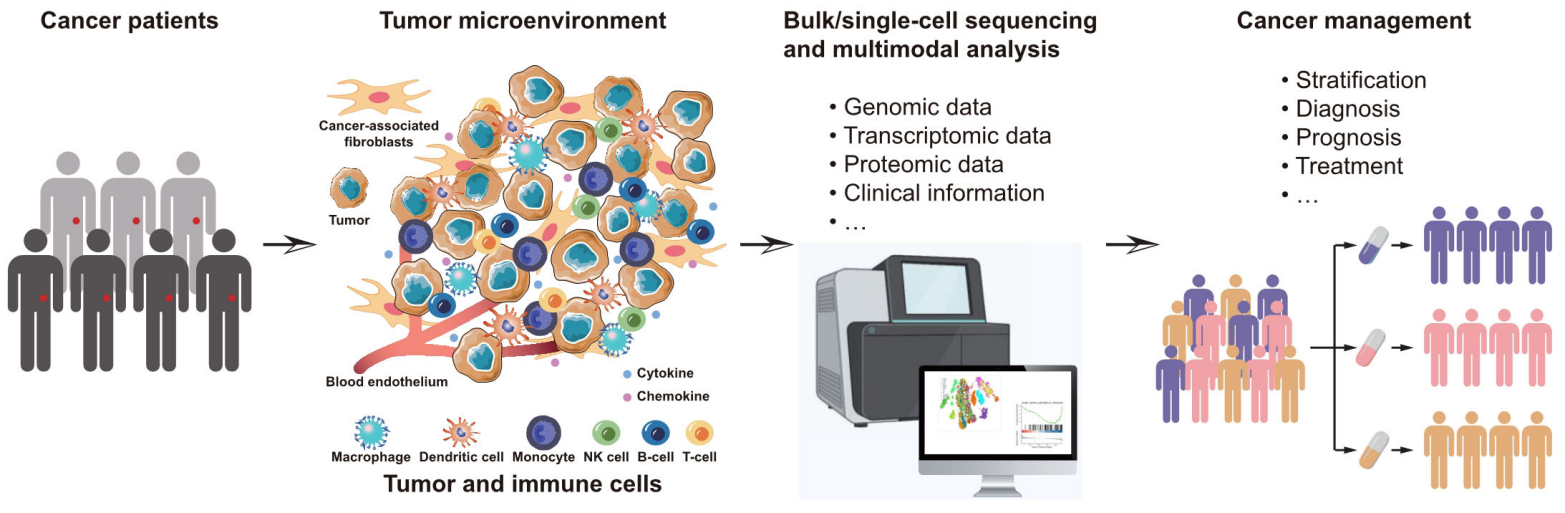
Schematic view of the aims for this Research Topic on Comprehensive profiling cancer immunity with multimodal approaches for clinical management.

Based on the publicly accessible single-cell datasets from immune checkpoint inhibitors (ICIs) regimens, Li et al. identified an MPR-expanding T cells meta-cluster (MPR-E) within the tumor microenvironment, characterized by augmented STAT5-ADGRE5 axis activity in stem-like CD8+ T cells (survT) relative to non-responders or pre-treatment samples. They constructed an ADGRE5-centered Tsurv model applicable to diverse tumor types, which effectively recognized responder T cell profiles in non-small cell lung cancer, melanoma, and urothelial cancer among others. This study provided insights into the mechanisms underlying major pathologic response to anti-PD1 therapy and could benefit the development of more precise strategies to predict and enhance the efficacy of cancer immunotherapy. Nam et al. revealed that HER2 positivity in non-muscle invasive bladder cancer (NMIBC) predicted a less favorable response to Bacillus Calmette-Guerin (BCG) therapy, which was associated with an aggressive tumor microenvironment characterized by increased PD-L1+ cells and other immune markers. They suggested that HER2 status may be linked to genetic traits more common in older individuals, potentially providing a basis for predicting recurrence and BCG treatment response. These findings supported the exploration of combining BCG with immune checkpoint inhibitors or HER2-targeted therapies for BCG-naive patients at high risk of treatment failure, particularly in the elderly.

Yoo et al. showed that the combination of stereotactic radiosurgery (SRS) and immunotherapy was promising in optimizing long-term control and overall survival rates for patients with brain metastases, challenging traditional treatment approaches. Preclinical and early-phase clinical trials suggest that SRS can stimulate antitumor immunity, and when integrated with immunotherapeutic agents, may enhance treatment outcomes for various intracranial metastases. Further studies are needed to determine optimal administration sequencing, radiation dosages, and fractionation regimens to maximize the synergistic effects of this combined therapy and to evaluate its safety and efficacy in different cancer types. Luo et al. described a case report that the use of Toripalimab, a novel ICI targeting the PD-1 receptor, in the treatment of advanced nasopharyngeal carcinoma, highlighting its efficacy in achieving complete remission but also emphasizing the risk of severe immune-related adverse events, particularly colitis and cytomegalovirus (CMV) infection. The report underscored the complexity of managing immune-related colitis induced by Toripalimab. It advocated the importance of vigilant monitoring and standardized biopsy procedures to manage and diagnose such complications effectively. The real-world retrospective study conducted by Wu et al. revealed that neoadjuvant tislelizumab combined with chemotherapy had a high objective response rate and major pathological response in patients with locally advanced oral or oropharyngeal squamous cell carcinoma (LAOOPSCC). They found that patients achieving major pathological responses had significantly better overall and disease-free survival rates. The treatment approach proposed in this study could be feasible and safe, with the potential for organ preservation, although further research with larger cohorts and longer follow-up is necessary to confirm survival benefits. On the other hand, Qian et al. proposed DeepLION2, a novel deep learning framework that enhanced the prediction of cancer-associated T cell receptors (caTCRs) by employing a combination of multi-instance contrastive learning and attention mechanisms focused on motifs. They showed that DeepLION2 significantly outperformed existing methods in accuracy, sensitivity, specificity, and area under the curve (AUC) across diverse cancer types. DeepLION2 could effectively identify key caTCRs and their crucial motifs, which are essential for TCR-peptide binding and hold promise for cancer detection and personalized immunotherapy. This study demonstrated the potential of DeepLION2 in advancing cancer research by providing a more accurate prediction of caTCRs and repertoires from TCR sequencing data. Additionally, Wang et al. systematically reviewed the intertwined nature of tuberculosis (TB) and tumor incidence, highlighting their mutual influence and impact on global health, with TB serving as a risk factor for tumor development and tumors increasing the likelihood of TB reactivation. They focused on the shared pathological mechanisms underlying TB and tumor comorbidity (TCWT), and summarized the recent advancements in mechanism-based diagnosis and treatments. Considering the complex interplay between TB and tumors, integrated and personalized treatment approaches are crucial for TCWT patients. This review emphasized the need for innovative diagnostic technologies and therapeutic strategies, including single-cell analysis, proteomics, cell therapy, and nanotechnology.

## Summary and perspective

Collectively, the studies published in this Research Topic could facilitate our understanding of cancer immunity, holding the potential to effectively enhance the clinical management of cancer. Recent advancements in multimodal profiling technologies have significantly enhanced the scientific community’s ability to attain a more structured and mechanistic understanding of the immune landscape and functional attributes within tumors (7, 8). Despite these developments, a critical knowledge gap persists concerning the identification and characterization of emergent immune-related pathways and biomarkers, alongside the elucidation of their underlying molecular mechanisms (9, 10). Addressing this problem is crucial to further enhance the precision and efficacy of cancer clinical management and therapeutic targeting (11). Therefore, the continued exploration of these untapped aspects of cancer immunity represents an urgent imperative in contemporary oncological research.

## Author contributions

GC: Writing – original draft, Writing – review & editing. YS: Writing – review & editing. WX: Writing – review & editing. DK: Writing – review & editing.
